# Philometrids (Nematoda: Philometridae) in carangid and serranid fishes off New Caledonia, including three new species

**DOI:** 10.1051/parasite/2014022

**Published:** 2014-05-19

**Authors:** František Moravec, Jean-Lou Justine

**Affiliations:** 1 Institute of Parasitology, Biology Centre of the Academy of Sciences of the Czech Republic, Branišovská 31 370 05 České Budějovice Czech Republic; 2 ISYEB, Institut Systématique, Évolution, Biodiversité, UMR7205 CNRS, EPHE, MNHN, UPMC, Muséum National d’Histoire Naturelle, CP51, 57 55 Rue Buffon 75231 Paris cedex 05 France

**Keywords:** Parasitic nematode, *Philometra*, Marine fish, *Alepes*, *Epinephelus*, *Selar*, New Caledonia

## Abstract

A recent examination of newly obtained specimens of philometrid nematodes (Philometridae) parasitising carangid and serranid fishes off New Caledonia, South Pacific, revealed the presence of several nematodes of the genus *Philometra* Costa, 1845, including three new species: *P*. *austropacifica* n. sp. (males and females) from the ovary of *Alepes vari* (Carangidae), *P*. *piscaria* n. sp. (males) from the ovary of *Epinephelus coioides* (Serranidae), and *P*. *selaris* n. sp. (males) probably from the abdominal cavity (found in washings) of *Selar crumenophthalmus* (Carangidae). The new species are characterised mainly by the length and structure of the spicules and gubernaculum, body size, their location in the host and the type of host. *Philometra austropacifica* n. sp. is the first known nominal gonad-infecting species of *Philometra* parasitising a carangid fish. In addition, the gravid female of *P*. *fasciati* Moravec & Justine, 2008 from the ovary of *Epinephelus fasciatus* (Serranidae) is described for the first time. Carangid host fish were identified by both morphology and DNA barcoding.

## Abstract (fr)

Une étude récente de spécimens nouvellement obtenus de nématodes Philometridae parasitant des poissons Carangidae et Serranidae de Nouvelle-Calédonie, dans le Pacifique Sud, a révélé la présence de plusieurs nématodes du genre *Philometra* Costa, 1845, dont trois nouvelles espèces : *P. austropacifica* n. sp. (mâles et femelles) de l’ovaire de *Alepes vari* (Carangidae), *P. piscaria* n. sp. (mâles) de l’ovaire d’*Epinephelus coioides* (Serranidae), et *P. selaris* n. sp. (mâles) probablement de la cavité abdominale (dans des lavages) de *Selar crumenophthalmus* (Carangidae). Les nouvelles espèces sont principalement caractérisées par la longueur et la structure des spicules et du gubernaculum, la taille du corps, par leur localisation dans l’hôte et par le type d’hôte. *Philometra austropacifica* n. sp. est la première espèce nominale connue de *Philometra* parasitant les gonades d’un Carangidae. En outre, la femelle gravide de *P. fasciati* Moravec et Justine, 2008 est décrite pour la première fois à partir de l’ovaire d’*Epinephelus fasciatus* (Serranidae). Les poissons hôtes Carangidae ont été identifiés à la fois par la morphologie et des codes-barres ADN.

## Introduction

Despite the report of ten species of *Philometra* Costa, 1845 from marine fishes in New Caledonian waters [17–20], our knowledge of the fauna of these important parasites in this region remains fragmentary. Recent studies carried out in adjacent waters off southern Indonesia and northern Australia by Moravec et al. [27], Dewi & Palm [2] and Moravec & Diggles [16] revealed the presence of several previously unknown philometrids in marine fishes, which may also occur in the New Caledonia region. Additional samples of philometrid nematodes, including three new species of *Philometra*, were collected from carangid and serranid perciform fishes. A taxonomic evaluation of this material is presented herein.

## Materials and methods

### Fish

Fishes were caught by line, speared or bought from the fish market; carangids from the fish market were taken with mackerel nets within a few miles off Nouméa, New Caledonia and were very fresh. In all cases the fishes came from locations within 50 km of Nouméa. All fish specimens were measured, weighed and photographed (except for *Epinephelus coioides*, see below). A unique number (JNC) was assigned to each fish. The parasitological material was then assigned a corresponding JNC linked to the respective fish host. Philometrid nematodes used in this study were recorded from the following four species of New Caledonian fishes: Carangidae: the herring scad, *Alepes vari* (Cuvier), and the bigeye scad, *Selar crumenophthalmus* (Bloch); Serranidae: the orange-spotted grouper, *Epinephelus coioides* (Hamilton), and the blacktip grouper, *E*. *fasciatus* (Forsskål). Since morphological identification of carangids is sometimes difficult, identification was confirmed by DNA barcoding. For *E*. *coioides*, we collected the material from fish ovaries sold at the fish market, other parts of the fish being sold separately. A discussion with the seller provided a reasonable certainty that all of the ovaries were from the same species; however, identification could not be ascertained by a direct examination of the fish.

### Molecular identification of fish

Fish DNA was extracted from tissue samples of three specimens using the NucleoSpin 96 tissue kit (Macherey-Nagel) following the manufacturer’s instructions. The 5′ region of the cytochrome oxidase I (COI) mitochondrial gene was amplified using the primers FishF1 (5′-TCAACCAACCACAAAGACATTGGCAC-3′) and FishR1 (5′-TAGACTTCTGGGTGGCCAAAGAATCA-3′) (Ward et al. 2005 [36]). The PCR reactions were performed in a 20-μL solution, containing 1 ng of DNA, 1 × CoralLoad PCR buffer, 3 mM MgCl2, 66 μM of each dNTP, 0.15 μM of each primer, and 0.5 units of Taq DNA polymerase (Qiagen). The amplification protocol was: 4 min at 94 °C, followed by 40 cycles of 94 °C for 30 s, 48 °C for 40 s, 72 °C for 50 s, with a final extension at 72 °C for 7 min. PCR products were purified and sequenced in both directions on 3730xl DNA Analyser 96-capillary sequencer (Applied Biosystems). Sequences were edited using CodonCode Aligner software (CodonCode Corporation, Dedham, MA, USA), compared with the GenBank database content using BLAST and deposited in GenBank under accession numbers KJ192344, KJ192345 and KJ192346. Species identification was confirmed using the BOLD identification engine [35]. The fish nomenclature adopted follows FishBase [3].

### Parasites

Nematodes were collected from fish examined with a wash method for intestines [6, 7]. Fish ovaries were separated, examined for large philometrid females, and scraped to look for smaller males.

The nematodes for morphological studies were fixed in hot 4% formaldehyde solution in physiological saline, or sometimes in hot 70% ethanol. For light microscopic examination, they were cleared with glycerine. Drawings were made with the aid of a Zeiss microscope drawing attachment. Specimens used for SEM were postfixed in 1% osmium tetroxide (in phosphate buffer), dehydrated through a graded acetone series, critical-point-dried and sputter-coated with gold; they were examined using a JEOL JSM-7401F scanning electron microscope at an accelerating voltage of 4 kV (GB low mode). All measurements are in micrometres unless otherwise indicated.

## *Philometra austropacifica* n. sp. (Figs. 1, 2)

urn:lsid:zoobank.org:act:F792D74F-CE4F-4B39-8C55-7B2847C62CD4

**Figure 1.**
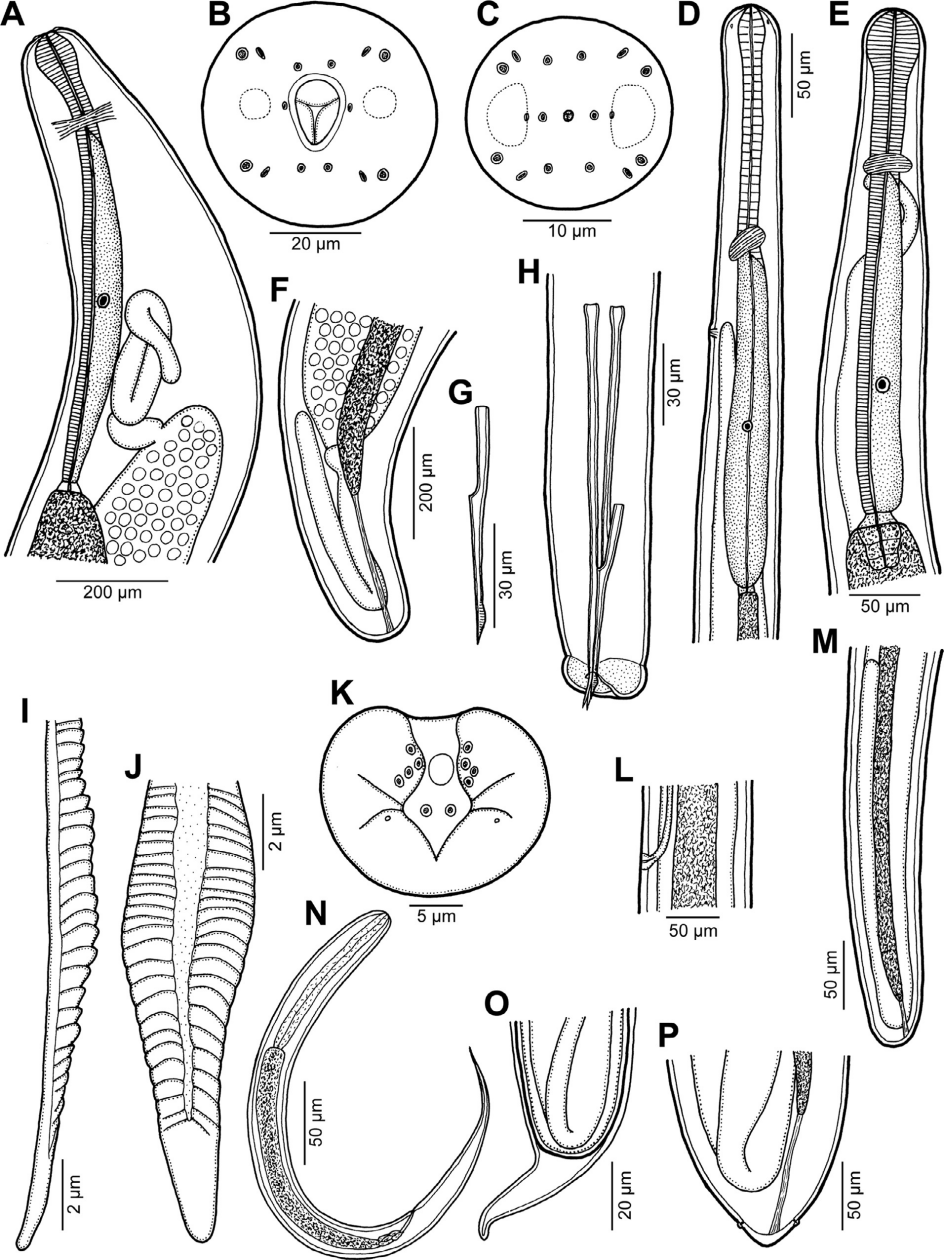
*Philometra austropacifica* n. sp. from *Alepes vari*. A: Anterior end of subgravid female, lateral view. B, C: Cephalic end of subgravid female and male, respectively, apical views. D: Anterior end of male, lateral view. E: Anterior end of mature female, lateral view. F: Posterior end of subgravid female, lateral view. G: Gubernaculum, lateral view. H: Posterior end of male, sublateral view. I, J: Distal end of gubernaculum, lateral and dorsal views. K: Caudal end of male, apical view. L: Vulva of mature female, lateral view. M: Posterior end of mature female, lateral view. N: Larva from uterus, lateral view. O: Caudal end of fourth-stage larva undergoing last moult. P: Caudal end of very small subgravid female, dorsoventral view.

**Figure 2.**
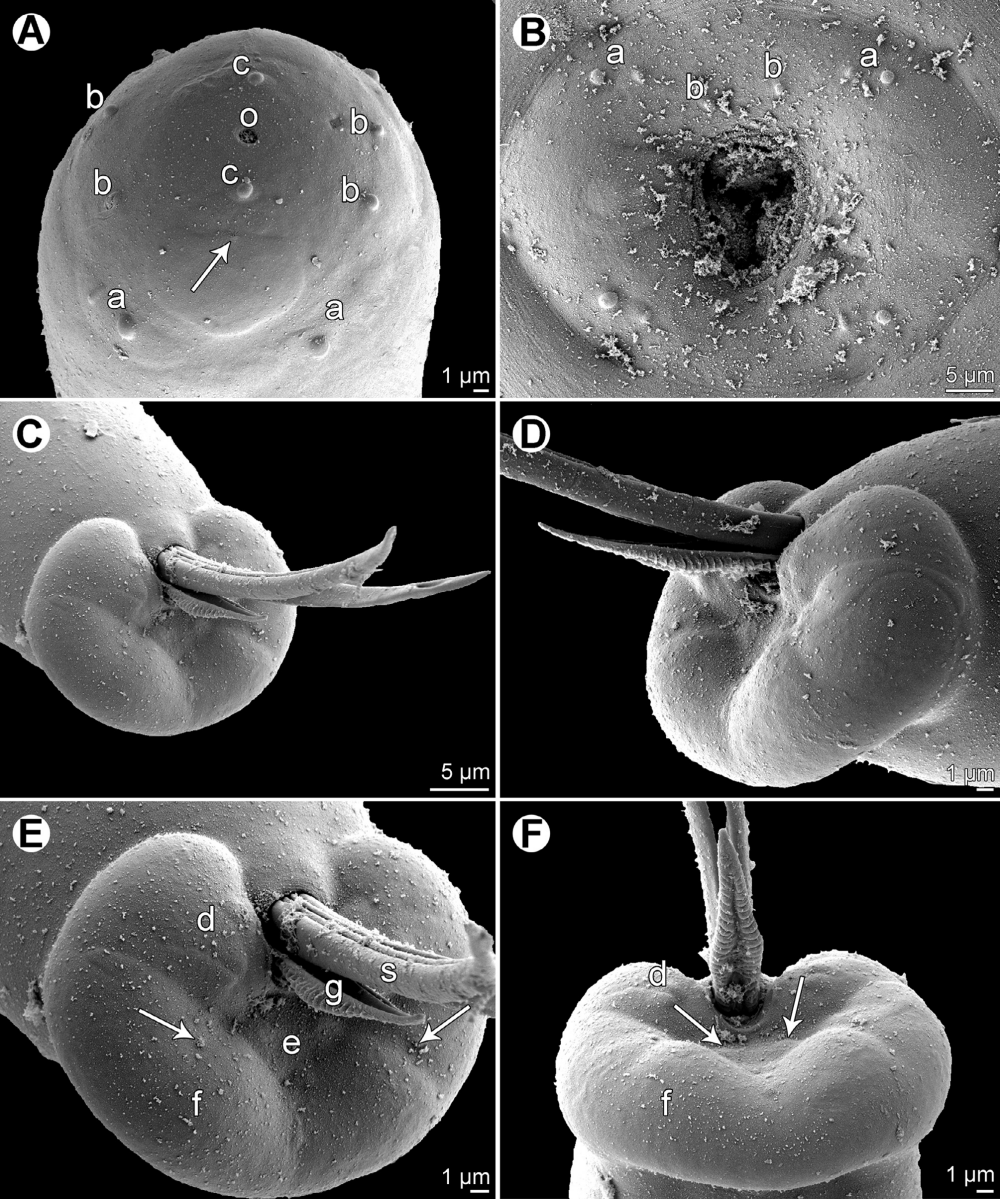
*Philometra austropacifica* n. sp. from *Alepes vari*, scanning electron micrographs. A: Cephalic end of male, subapical view (arrow indicates amphid). B: Cephalic end of subgravid female, apical view. C: Caudal end of male, subapical view (note aperture near distal tip of one spicule). D: Same, sublateral view (another specimen). E: Caudal end of male, subapical view (enlarged; arrows indicate phasmids). F: Same, dorsal view (arrows indicate papillae situated posterior to cloacal aperture). *Abbreviations*: a, submedian pair of cephalic papillae of external circle; b, submedian cephalic papilla of internal circle; c, lateral cephalic papilla of internal circle; d, group of four caudal papillae near cloacal aperture; e, pair of papillae situated posterior to cloacal aperture; f, caudal mound; g, gubernaculum; o, oral aperture; s, spicule.

Type-host: Herring scad, *Alepes vari* (Cuvier) (Carangidae, Perciformes) (fork length 290–310 mm). Molecular confirmation: our sequence KJ192344 for fish JNC3104 had a 100% match with sequences in the BOLD identification engine (24-01-2014).

Site of infection: Ovary.

Type locality: Fish market, Nouméa City, New Caledonia. Fish were taken with mackerel nets within a few miles off Nouméa. Holotype JNC3104, date 19 November 2009; paratypes JNC3128, date 25 November 2009.

Prevalence and intensity: two fish infected/3 fish with ovaries examined); 6 and 11 nematode specimens.

Type specimens: Holotype, allotype and seven paratypes in the Muséum National d’Histoire Naturelle, Paris (MNHN JNC 3104A, JNC 3104B, JNC 3128A) and five paratypes in the Helminthological Collection of the Institute of Parasitology, Biology Centre of the Academy of Sciences of the Czech Republic, České Budějovice (Cat. No. N–1041).

Etymology: The specific name of this nematode is a Latin adjective composed of the words *australis* (= southern) and *Pacificus* (= Pacific), which relates to the region of the occurrence of this parasite, that is South Pacific.

### Description

*Male* (eight specimens; measurements of holotype in parentheses): body whitish, filiform, tapering to both ends, 1.47–2.73 (2.73) mm long; maximum width at middle 48–69 (69); anterior part of body slightly narrower just posterior to cephalic end (Fig. 1D); body width at this narrowed part 27–36 (33). Maximum width/body length 1:27–43 (1:40); width of cephalic end 33–39 (36), that of posterior end 30–33 (36). Cuticle smooth. Cephalic end rounded. Oral aperture small, oval, surrounded by 14 cephalic papillae arranged in two circles: external circle formed by four submedian pairs of papillae (each pair consisting of one circular and one narrower, more elongate papilla); internal circle formed by four submedian and two lateral papillae (Figs. 1C, 2A). Small lateral amphids just posterior to lateral papillae of internal circle (Figs. 1C, 2A). Oesophagus readily visible, 270–387 (381) long, maximum width 15–27 (27), comprising 14–23 (14)% of body length, slightly inflated at anterior end; posterior part of muscular oesophagus overlapped by well-developed oesophageal gland with large cell nucleus in middle (Fig. 1D); anterior oesophageal inflation 27–36 (27) long and 18–24 (21) wide. Nerve ring, excretory pore and oesophageal nucleus 111–150 (150), 138–210 (210) and 204–273 (273), respectively, from anterior extremity. Testis reaches anteriorly almost to level of nerve ring. Posterior end of body blunt, with broad, V-shaped mound extending dorsally and laterally (Figs. 1H, K, 2C–F). Four pairs of very flat, indistinct caudal papillae close to each other situated on sides of cloacal aperture on mound; one additional pair of papillae located posterior to cloacal aperture between both lateral arms of mound (Figs. 1K, 2C–D). Pair of small phasmids present at about middle of each mound arm (Figs. 1K, 2E). Spicules slender, needle-like, equal or slightly subequal, with somewhat expanded proximal and sharply pointed distal tips (Figs. 1H, 2C–F); length of spicules 153–174 and 150–171 (165 and 168), comprising 6–11 (6)% of body length. Length ratio of spicules 1:1.00–1.06 (1:1.00). Gubernaculum narrow, 66–84 (81) long, with anterior portion slightly bent dorsally; length of anterior bent part 27–36 (30), representing 36–43 (37)% of entire gubernaculum length; posterior end of gubernaculum with dorsal protuberance composed of two longitudinal parts bearing numerous transverse lamella-like structures and demarcating depressed smooth field between them (Figs. 1G–J, 2C–F). Length ratio of gubernaculum and spicules 1:2.04–2.32 (1:2.04). Spicules and gubernaculum well sclerotised, yellowish but with anterior part of gubernaculum colourless.

*Gravid female* (single fragment of middle part of one specimen): body of fragment brownish, with distinct dark-brown intestine visible through cuticle and many larvae and eggs in uterus; length of body fragment about 15 mm, maximum width 680. Larvae from uterus 339–414 long, maximum width 15–18; length of oesophagus 99–126 (27–31% of body length), of tail 96–114 (25–29%).

*Subgravid female* (two small complete specimens and four body fragments of larger specimens; measurements of allotype in parentheses): body of fixed specimens whitish to brownish, filiform, with rounded ends; posterior part of body narrower than anterior part. Body length of complete specimens 9.86 and (11.19) mm, lengths of body fragments 9.33–13.60 mm; maximum width 204–408 (204). Cuticle smooth. Maximum width/body length in complete specimens 1:40 and (1:55). Width of cephalic end 109–136 (109). Cephalic papillae small, indistinct when viewed laterally (Fig. 1A). Oral aperture dorsoventrally oval with broader dorsal part, surrounded by outer circle of four pairs of submedian cephalic papillae and inner circle of six single papillae (two lateral and four submedian); each outer submedian pair formed by distinctly larger circular papilla and smaller elongate papilla (Figs. 1B, 2B). Amphids indistinct. Bottom of mouth formed by lobes of three oesophageal sectors (Figs. 1B, 2B). Oesophagus including moderately developed anterior bulbous inflation 571–925 (639) long, comprising (5.7%) and 5.8% of body length in complete specimens; anterior inflation 78–99 (90) long and 66–81 (75) wide; maximum width of posterior part of oesophagus including gland 54–82 (82). Oesophageal gland well developed, opens into oesophagus just posterior to nerve ring, with large cell nucleus (Fig. 1A). Nerve ring and oesophageal nucleus 177–204 (177) and 408–585 (585), respectively, from anterior extremity. Small ventriculus 12–18 (18) long and 39–68 (42) wide. Posterior end of intestine attached by ligament ventrally to body wall near its caudal end (Figs. 1F, P); ligament 150–180 (150) long. Vulva and anus absent. Ovaries reflexed, situated near body ends (Figs. 1A, F). Uterus filled with numerous mature or immature eggs. Posterior end of larger specimens rounded, 136 wide, without any caudal projections (Fig. 1F); that of two smallest complete specimens somewhat cone-shaped, 109 (109) wide, with pair of minute, indistinct lateral caudal projections (Fig. 1P).

*Nongravid female* (three mature specimens): body length 2.06–4.26 mm; maximum width 60–120; maximum width/length ratio 1:34–37. Width of anterior end 42–57, of posterior end 27–48. Entire oesophagus 405–565 long and 18–51 wide. Anterior oesophageal bulb 39–48 long, 33–42 wide. Nerve ring and oesophageal nucleus 108–129 and 279–324, respectively, from anterior extremity (Fig. 1E). Intestinal ligament 57–99 long. Vulva and incompletely developed vagina present only in two smaller specimens 2.06 and 3.14 mm long; former situated 2.03–2.11 mm from anterior extremity (at 54–64% of body length) (Fig. 1L). Uterus empty. Caudal end rounded (Fig. 1M).

*Fourth-stage larva* (one female larva undergoing last moult): body length 1.95 mm, maximum width 51; maximum width/length ratio 1:38. Width of anterior end 33, of posterior end 30. Entire oesophagus 315 long and 24 wide. Anterior oesophageal bulb 36 long, 24 wide. Nerve ring and oesophageal nucleus 108 and 216, respectively, from anterior extremity. Intestinal ligament 60 long. Vulva situated 1.35 mm from anterior extremity (at 69% of body length). Uterus empty. Caudal end rounded, still inside shed cuticle (Fig. 1O); length of shed cuticular exuviae 39.

### Discussion

Of the 16 gonad-infecting species of *Philometra* described with lamella-like structures on the gubernaculum [13], a distinct dorsal protuberance near the distal extremity of the gubernaculum, which occurs in the new species, is only present in *P*. *gerrei* Moravec & Manoharan, 2013, *P*. *johnii* Moravec & Ali, 2013, *P*. *lateolabracis* (Yamaguti, 1935), *P*. *lopholatili* Moravec & Bakenhaster, 2013, *P*. *otolithi* Moravec & Manoharan, 2013, *P*. *priacanthi* Moravec & Justine, 2009 and *P*. *terapontis* Moravec, Gopalakrishnan, Rajkumar, Saravanakumar & Kaliyamoorthy, 2011 [10, 13, 14, 19, 22, 23, 32]. However, the protuberance consisting of two dorsolateral lamellar parts separated from each other by a smooth median field, as shown to occur in the new species, is known only in *P*. *johnii*, from *Johnius dussumieri* (Cuvier) (Sciaenidae) in the Persian Gulf and off northern Australia [10, 16], *P*. *otolithi*, from *Otolithes ruber* (Bloch & Schneider) (Sciaenidae) in the Persian Gulf, the Sea of Oman and off India [21–23], and *P*. *lopholatili*, from *Lopholatilus chamaeleonticeps* Goode & Bean (Malacanthidae) in the Gulf of Mexico [13].

The morphology of all these three species is similar to that of *P*. *austropacifica* n. sp. However, in contrast to the new species, the male caudal mound of *P*. *johnii* and *P*. *otolithi* is distinctly dorsally interrupted (vs. non-interrupted) and the distal lamellar tip of their gubernaculum is somewhat broader and shorter, with lamellae extending posteriorly to the smooth dorsal field and almost to the gubernaculum extremity (vs. lamellae extending posteriorly only as far as the level of the end of the smooth field). They also differ in the host family (Sciaenidae vs. Carangidae).

The shape of the male caudal mound and the structure of the distal tip of the gubernaculum in *P*. *lopholatili* are similar to those of the new species, but the males of *P*. *lopholatili* are distinctly longer (3.26–3.86 vs. 1.47–2.73 mm), with a more elongate and longer oesophagus (510–738 vs. 270–387 μm) and a distinctly longer gubernaculum (114–126 vs. 66–84 μm). The lengths of spicules of both species overlap (165–189 μm in *P*. *lopholatili* and 150–171 μm in *P*. *austropacifica*), but they represent only 4–5% of the body length in *P*. *lopholatili* and 6–11% in that of *P*. *austropacifica*. The gubernaculum/spicules length ratio is also different in these two species (1:1.37–1.50 vs. 1:2.04–2.32 in the new species). Both species also differ in the host family (Malacanthidae vs. Carangidae) and the geographical distribution (North Atlantic vs. South Pacific).

Fishes of the Carangidae are frequently found to be infected with philometrid species of *Buckleyella* Rasheed, 1963, *Caranginema* Moravec, Montoya-Mendoza et Salgado-Maldonado, 2008, *Philometra* Costa, 1845 and *Philometroides* Yamaguti, 1935, which parasitise different organs of their hosts [9]. Of the eight identified philometrid species reported from carangid fishes, only two, *Philometra globiceps* (Rudolphi, 1819) and *P*. *lateolabracis* (Yamaguti, 1935), were found in the gonads of *Alepes djedaba* (Forsskål) (as *Caranx kalla*) and *Seriola dumerili* (Risso), respectively [9, 31]; other species are found in the abdominal cavity, eyes, musculature or subcutaneous tissues of their fish hosts. However, as pointed out by Quiazon et al. [33] and Moravec & Ali [10], these two species were evidently misidentified. Consequently, *P*. *austropacifica* n. sp. is the first known nominal gonad-infecting species of *Philometra* parasitising a carangid fish.

## *Philometra piscaria* n. sp. (Figs. 3, 4)

urn:lsid:zoobank.org:act:44885F1B-4287-4E9F-8557-078ACCE1D6CC

**Figure 3.**
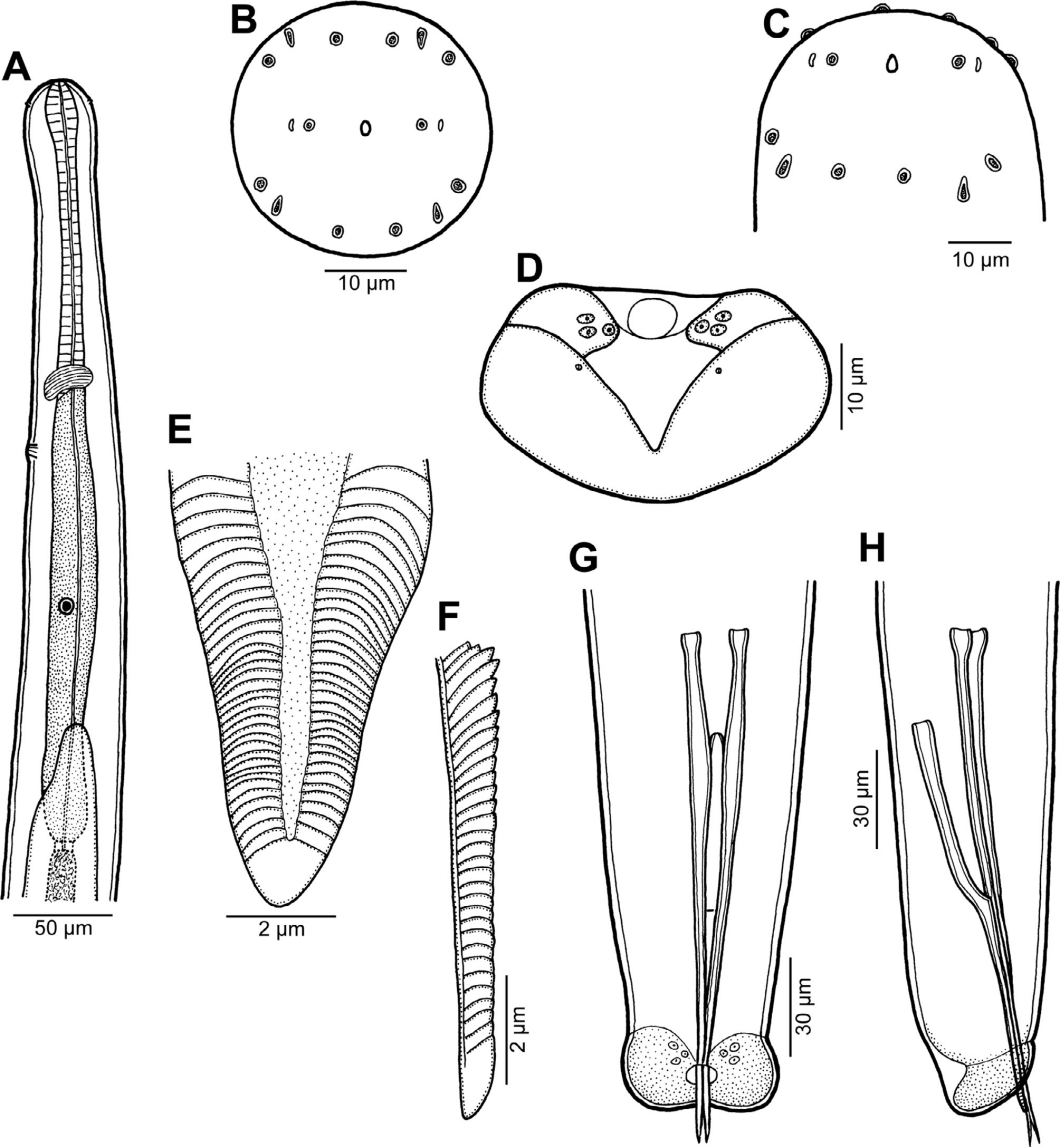
Male of *Philometra piscaria* n. sp. from *Epinephelus coioides*. A: Anterior end of body, lateral view. B, C: Cephalic end, apical and subdorsoventral views. D: Caudal end, apical view. E, F: Distal end of gubernaculum, dorsal and lateral views. G, H: Posterior end of body, ventral and lateral views.

**Figure 4.**
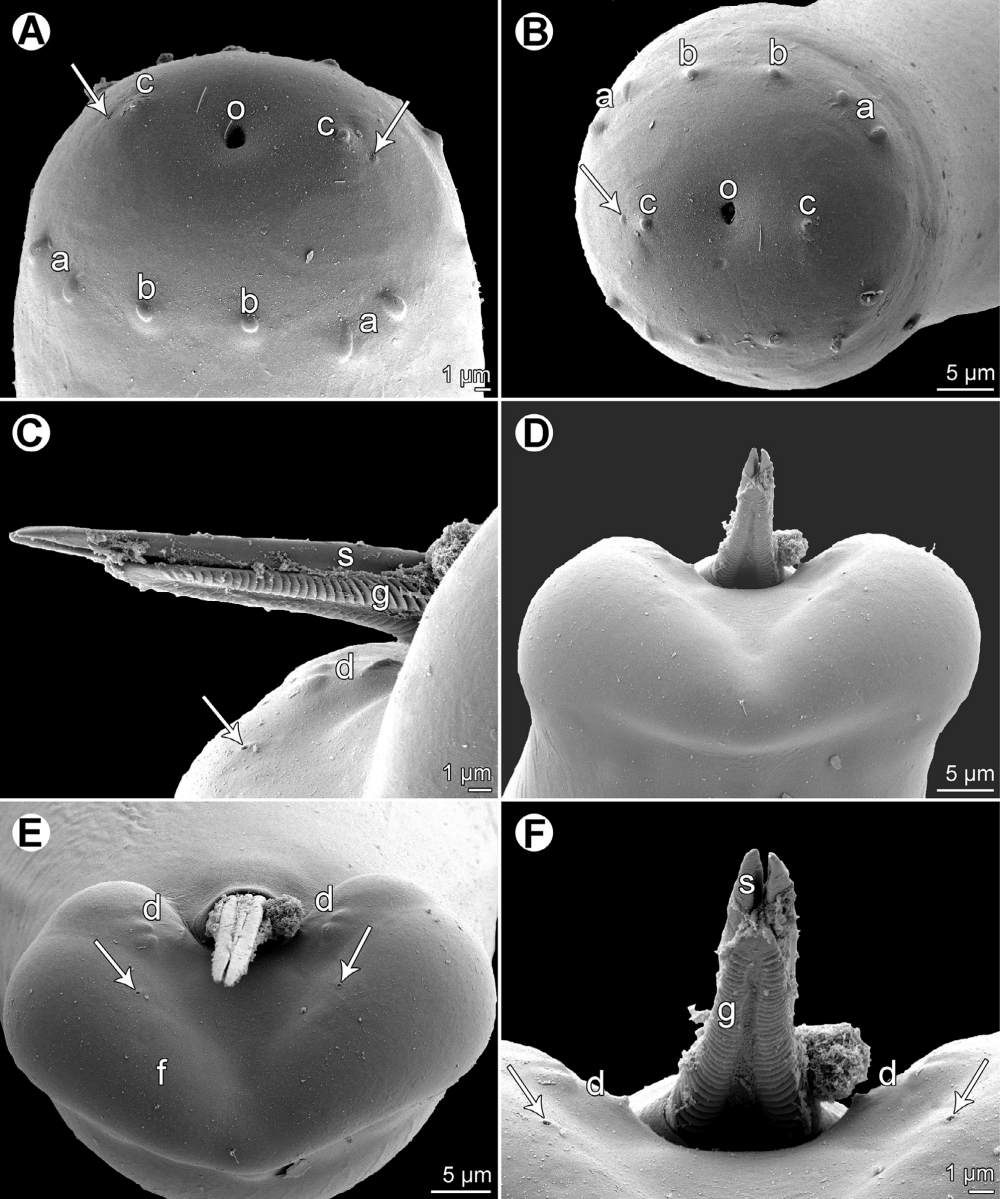
*Philometra piscaria* n. sp. from *Epinephelus coioides*, scanning electron micrographs of male. A, B: Cephalic end, subapical and apical views (arrows indicate amphids). C: Distal ends of gubernaculum and spicules, lateral view (arrow indicates phasmid). D, E: Caudal end, dorsal and apical views, respectively (arrows indicate phasmids). F: Region of cloacal aperture, dorsal view (arrows indicate phasmids). *Abbreviations*: a, submedian pair of cephalic papillae of external circle; b, submedian cephalic papilla of internal circle; c, lateral cephalic papilla of internal circle; d, group of three caudal papillae near cloacal aperture; f, caudal mound; g, gubernaculum; o, oral aperture; s, spicule.

Type host: Probably the orange-spotted grouper, *Epinephelus coioides* (Hamilton) (Serranidae, Perciformes). The specimens were collected from ovaries sold separately at the fish market; however, we had good reason to believe that the fish was *E*. *coioides*.

Site of infection: Ovary.

Type locality: Fish market, Nouméa City, New Caledonia. Date: 30 October 2009.

Prevalence and intensity: Prevalence unknown; five nematode specimens. Twenty whole ovaries were examined at the fish market the same day and female philometrids were visible in none of them. Male philometrids were found in 1/1 ovary macerated and examined under a binocular microscope.

Type specimens: Holotype and one paratype in the Muséum National d’Histoire Naturelle, Paris (MNHN JNC 3082) and one paratype in the Helminthological Collection of the Institute of Parasitology, Biology Centre of the Academy of Sciences of the Czech Republic, České Budějovice (Cat. No. N–1040).

Etymology: The specific name *piscaria* is an adjective derived from the Latin name for a fish market, that is (*taverna*) *piscaria*, and relates to the place where specimens of this nematode species were obtained.

### Description

*Male* (five specimens; measurements of holotype in parentheses): body whitish, filiform, tapering to both ends, 3.67–4.19 (4.11) mm long, maximum width at middle 66–75 (75); anterior part of body slightly narrower just posterior to cephalic end (Fig. 3A); body width at this narrowed part 36–42 (42). Maximum width/body length 1:51–61 (1:55); width of cephalic end 39–45 (45), that of posterior end 33–36 (36). Cuticle smooth. Cephalic end rounded. Oral aperture small, oval, surrounded by 14 cephalic papillae arranged in two circles: external circle formed by four submedian pairs of papillae (each pair consisting of one circular and one narrower, more elongate papilla); internal circle formed by four submedian and two lateral papillae (Figs. 3B, C, 4A, B). Small lateral amphids just posterior to lateral papillae of internal circle (Figs. 3B, C, 4A, B). Oesophagus readily visible, 531–615 (555) long, maximum width 27–33 (27), comprising 14–16 (14)% of body length, slightly inflated at anterior end; posterior part of muscular oesophagus overlapped by well-developed oesophageal gland with large cell nucleus in middle (Fig. 3A); anterior oesophageal inflation 33–36 (36) long and 12–18 (15) wide. Nerve ring, excretory pore and oesophageal nucleus 135–195 (195), 222–252 (252) and 351–408 (408), respectively, from anterior extremity. Testis reaches as far anteriorly as about midway between oesophageal nucleus and oesophago-intestinal junction (Fig. 3A). Posterior end of body blunt, with broad, V-shaped mound extending dorsally and laterally (Figs. 3D, G, H, 4D, E), forming short tail 18–24 (24) long (Fig. 3H). Three pairs of very flat, indistinct caudal papillae situated close to each other at sides of cloacal aperture on mound (Figs. 3D, G, 4C–F). Pair of small phasmids present at about middle of each mound arm (Figs. 3D, 4C, E, F). Spicules slender, needle-like, equal or slightly subequal, with somewhat expanded proximal and sharply pointed distal tips (Figs. 3G, H, 4C–F); length of spicules 174–180 and 171–180 (180), comprising 4–5 (4)% of body length. Length ratio of spicules 1:1.00–1.02 (1:1.00). Gubernaculum narrow, 126–144 (132) long, with anterior portion somewhat bent dorsally; length of anterior bent part 57–72 (57), representing 43–57 (43)% of entire gubernaculum length; posterior end of gubernaculum with dorsal protuberance composed of two longitudinal parts bearing numerous transverse lamella-like structures and demarcating depressed smooth field between them (Figs. 3E, F, H, 4C, D, F). Length ratio of gubernaculum and larger spicule 1:1.26–1.45 (1:1.36). Proximal ends of spicules weakly sclerotised; spicules and gubernaculum yellowish, anterior part of gubernaculum colourless.

*Female*: Unknown.

### Discussion

To date, nine nominal gonad-infecting species of *Philometra* are known to parasitise fishes of the perciform family Serranidae: *P*. *charlestonensis* Moravec, de Buron, Baker & González-Solís, 2008, *P*. *cyanopodi* Moravec & Justine, 2008, *P*. *fasciati* Moravec & Justine, 2008, *P*. *hyporthodi* Moravec & Bakenhaster, 2013, *P*. *jordanoi* (López-Neyra, 1951), *P*. *managatuwo* Yamaguti, 1941, *P*. *margolisi* Moravec, Vidal-Martínez & Aguirre-Macedo, 1995, *P*. *mexicana* Moravec & Salgado-Maldonado, 2007 and *P*. *serranellicabrillae* Janiszewska, 1949 [13, 15]. Two of them, *P*. *cyanopodi* from *Epinephelus cyanopodus* (Richardson) and *P*. *fasciati* from *E*. *fasciatus* (Forsskål), were described from New Caledonian waters [17, 18].

The new species can be easily distinguished by the length of its spicules (171–180 μm) from *P*. *charlestonensis* (132–141 μm), *P*. *hyporthodi* (135–138 μm), *P*. *jordanoi* (about 260 μm), *P*. *margolisi* (432–468 μm), *P*. *mexicana* (90–120 μm) and *P*. *fasciati* (147–156 μm). The spicules of *P*. *cyanopodi* are only slightly longer (186–228 vs. 171–180 μm), but the males are shorter (2.72–3.59 vs. 3.67–4.19 mm); the length of spicules represents 6–8% of the body length in *P*. *cyanopodi*, as compared with 4–5% in *P*. *piscaria* n. sp. The male oesophagus of *P*. *cyanopodi* is longer (654–765 vs. 531–615 μm) and the proximal bent portion of the gubernaculum forms only 30–39% (vs. 43–57%) of its entire length. The males of *P*. *managatuwo* and *P*. *serranellicabrillae* are unknown, so at present these species can be distinguished from *P*. *piscaria* only by their geographical distribution (Mediterranean region and Japan vs. New Caledonia).

Two other philometrid species, *Philometra epinepheli* Dewi & Palm, 2013 and *Spirophilometra endangae* Dewi & Palm, 2013, were recently described based on females found in the opercula and fins, respectively, of *E*. *coioides* in the South Bali Sea, Indonesia [2]. Subsequently, the same two species were recorded in *E*. *coioides* off the northern coast of Australia [16], so it is highly probable that they also occur in this fish host in New Caledonian waters. Since the male of *P*. *epinepheli* remains unknown, as does the female of *P*. *piscaria*, for the time being it is impossible to distinguish these two species morphologically. However, individual species of *Philometra* are known to be characterised by their location in the host [5, 9], so these two species can be distinguished on the basis of their very different sites of infection (ovary vs. fins).

## *Philometra fasciati* Moravec & Justine, 2008 (Figs. 5–7)

urn:lsid:zoobank.org:act:15EA5EF7-0697-4ADC-91D1-5ED75B709460

**Figure 5.**
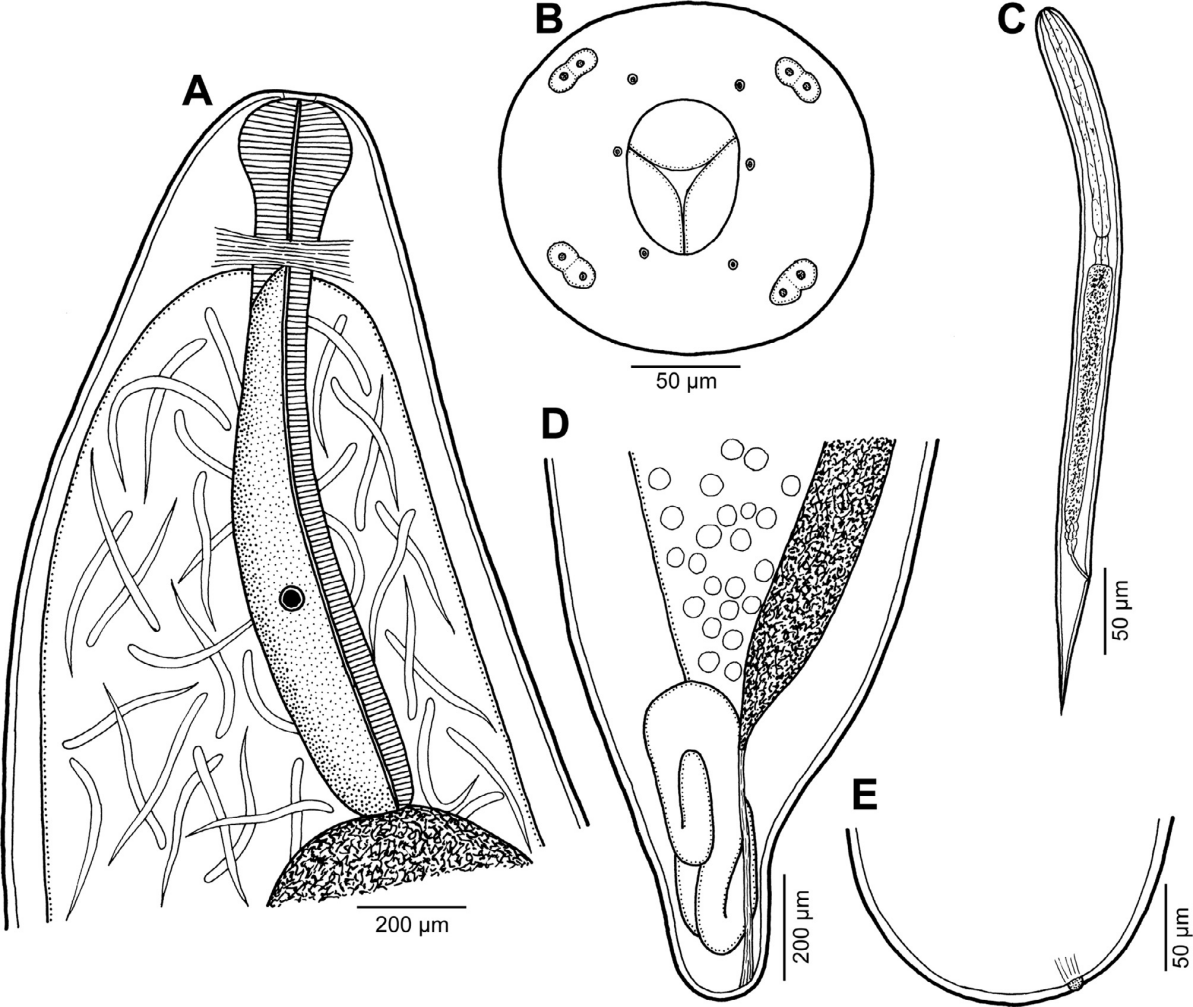
Gravid female of *Philometra fasciati* Moravec & Justine, 2008 from *Epinephelus fasciatus*. A: Anterior end, lateral view. B: Cephalic end, apical view. C. Larva from uterus, lateral view. D: Posterior end, lateral view. E: Caudal extremity, lateral view.

**Figure 6.**
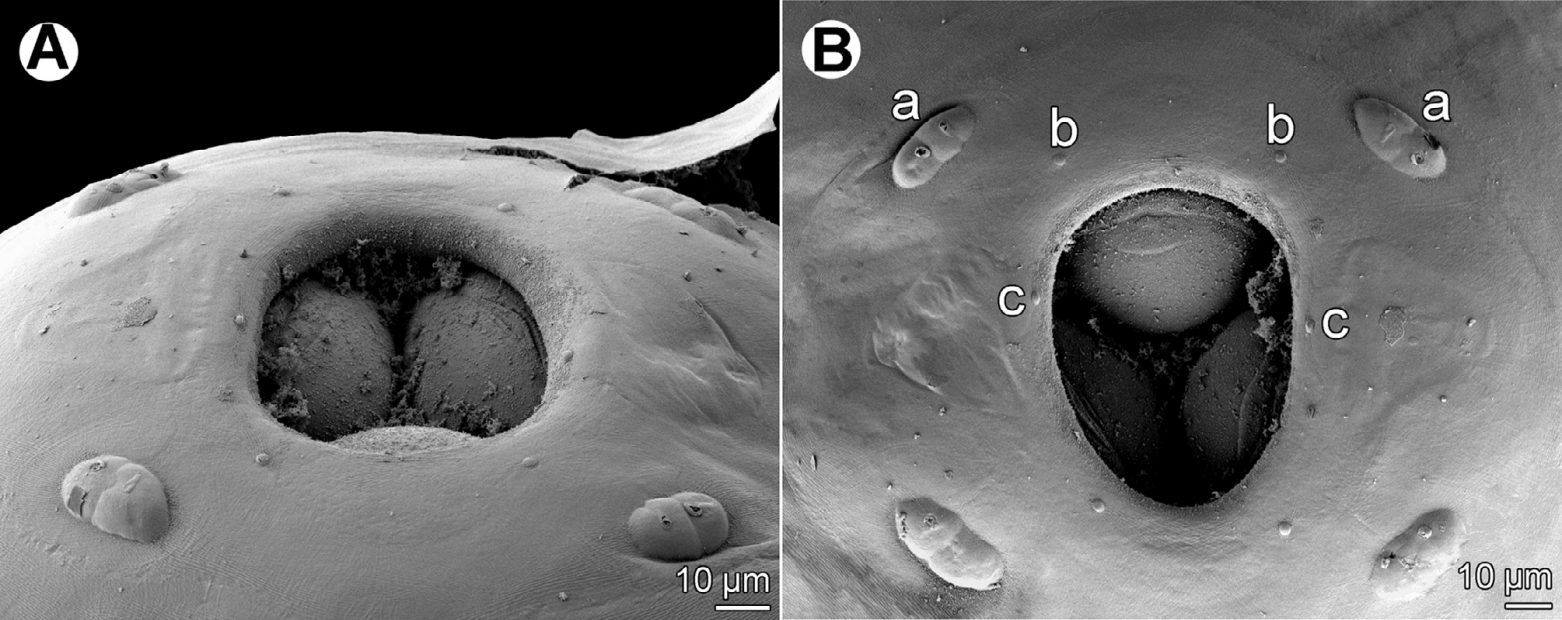
*Philometra fasciati* Moravec & Justine, 2008 from *Epinephelus fasciatus*, scanning electron micrographs of cephalic end of gravid female. A: Subdorsoventral view. B: Apical view. *Abbreviations*: a, double submedian papilla of external circle; b, submedian papilla of internal circle; c, lateral papilla of internal circle.

**Figure 7.**
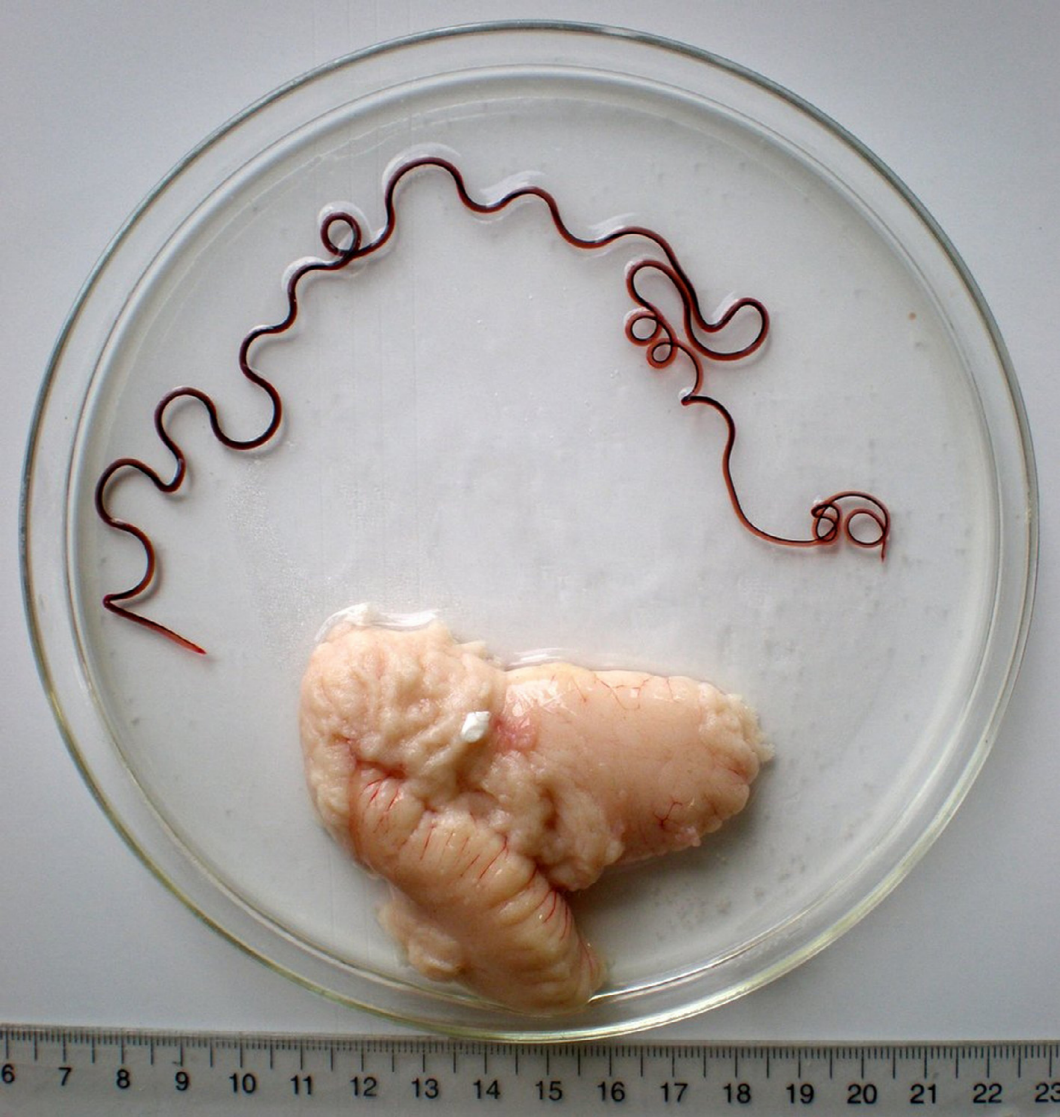
*Philometra fasciati* gravid female removed from the ovary of *E*. *fasciatus*. Scale: centimetres and millimetres.

Host: Blacktip grouper, *Epinephelus fasciatus* (Forsskål) (Serranidae, Perciformes) (fork length 262 mm).

Site of infection: Ovary.

Locality: Off Récif Kué (166°32′ E, 22°36′30″ S), Nouméa, New Caledonia, 9 December 2008.

Prevalence and intensity: Of 61 fish (both sexes) whose abdominal organs were examined (Justine et al. 2010), only one was found with a visible philometrid female in the ovary. Intensity: 1. Meticulous scraping of the ovary containing the female failed to detect any male.

Voucher: Muséum National d’Histoire Naturelle, Paris (MNHN JNC 2832).

### Description

*Gravid female* (one specimen): body of fixed specimen brownish (red when alive), with distinct dark-coloured intestine visible through cuticle, with rounded ends. Posterior part of body narrower than anterior part; maximum width in region posterior to oesophagus. Body 382 mm long, maximum width 1.63 mm, maximum width/body length ratio 1:234. Width of cephalic end 218. Cephalic papillae very small, indistinct when viewed laterally (Fig. 5A). Oral aperture large, oval, surrounded by four submedian double cephalic papillae of external circle and six single papillae (two lateral and four submedian) of internal circle. Amphids indistinct. Base of mouth formed by lobes of three oesophageal sectors (Figs. 5B, 6A, B). Oesophagus including anterior bulbous inflation 1.58 mm long, representing 0.4% of body length; bulb well developed, 231 long and 231 wide; maximum width of oesophagus including gland 204. Oesophageal gland well developed, opens into oesophagus just posterior to nerve ring, with large cell nucleus in middle. Nerve ring and oesophageal nucleus 340 and 952, respectively, from anterior extremity. Ventriculus indistinct. Intestine wide at anterior end; its posterior end narrow, attached by short ligament ventrally to body wall near caudal end; ligament 544 long. Vulva and anus absent. Ovaries relatively short, thick, reflected, situated near body ends. Uterus occupies most of body space, reaching to level of nerve ring, filled with numerous larvae 369–561 long and 21–27 wide; length of oesophagus 126–180, of tail 69–102, representing 32–36% and 17–19%, respectively, of entire body length of larva (Fig. 5C). Posterior end of female narrows abruptly in region of ligament, rounded, 204 wide, with 2 minute, indistinct subterminal papilla-like projections (Figs. 5D, E).

### Discussion

*Philometra fasciati* was established by Moravec & Justine [18] based on their earlier description of the male nematodes found in the ovary of *Epinephelus fasciatus* off New Caledonia and originally considered to be *P*. *lateolabracis* [17]. The correct identification of these nematodes was only possible after the description of the previously unknown male of *P*. *lateolabracis* by Quiazon et al. [32].

As mentioned above, *P*. *fasciati* is known only from males and a single small mature female 3.16 mm long [17]. The present description of the conspecific gravid female extends our knowledge of the morphology of this species considerably. Among gonad-infecting species of *Philometra* parasitising serranid fishes, which have gravid females of <200 mm in length in most species, *P*. *fasciati* is the second largest (384 mm), being longer (445 mm) only in material identified as *P*. *managatuwo* by Moravec et al. [26] from *Epinephelus septemfasciatus* (Thunberg) off Japan. The gravid female of *P*. *fasciati* differs from those of other gonad-infecting species of *Philometra* in that its paired submedian cephalic papillae of the external circle are fused together to form large, somewhat elevated double papillae, in contrast to the well-separated papillae of other species.

## *Philometra selaris* n. sp. (Figs. 8, 9)

urn:lsid:zoobank.org:act:2E87C1FD-6638-4B9D-BD06-4D99597B9795

**Figure 8.**
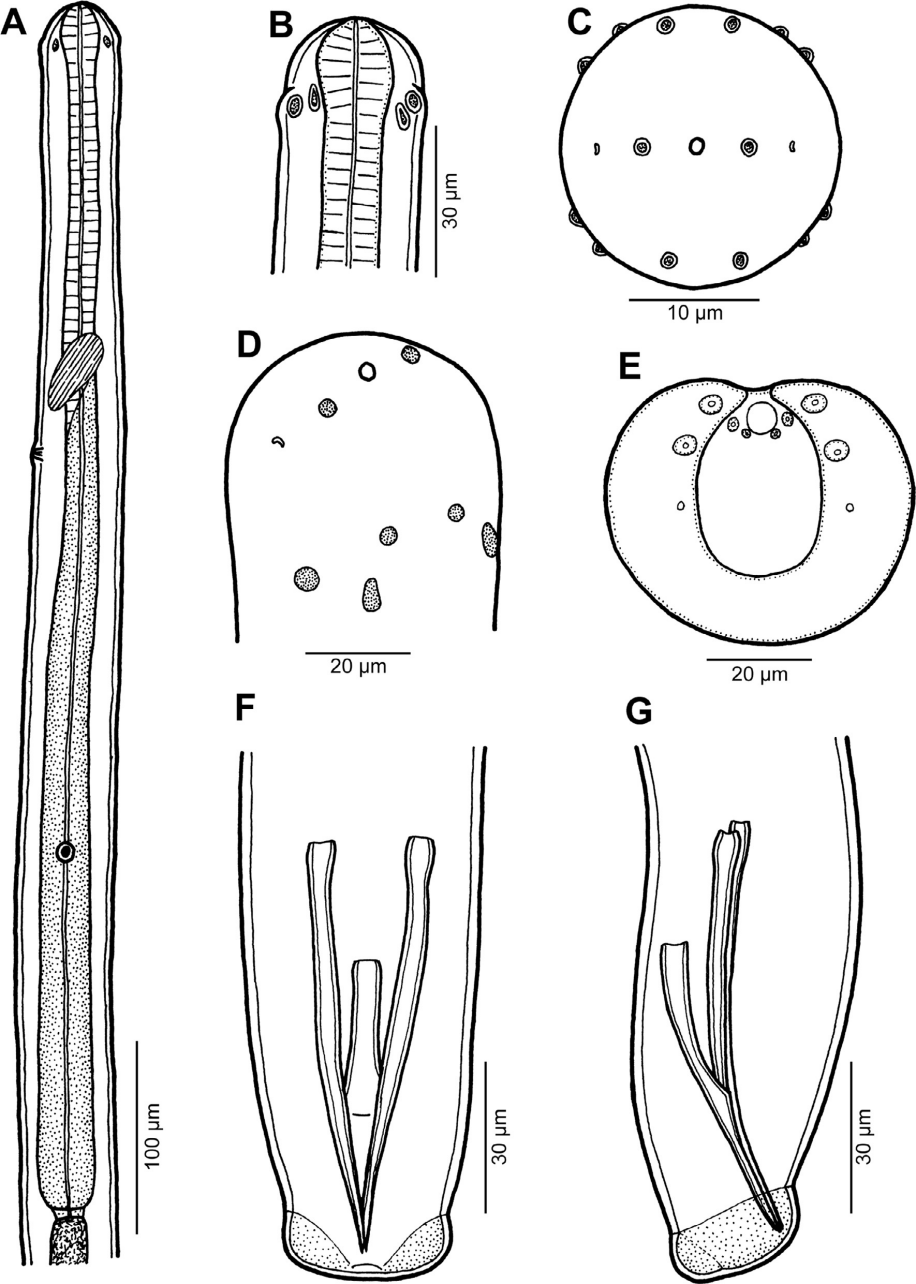
Male of *Philometra selaris* n. sp. from *Selar crumenophthalmus*. A: Anterior end of body, lateral view. B, C, D: Cephalic end, lateral, apical and sublateral views, respectively. E: caudal end, apical view. F, G: Posterior end, ventral and lateral views, respectively.

**Figure 9.**
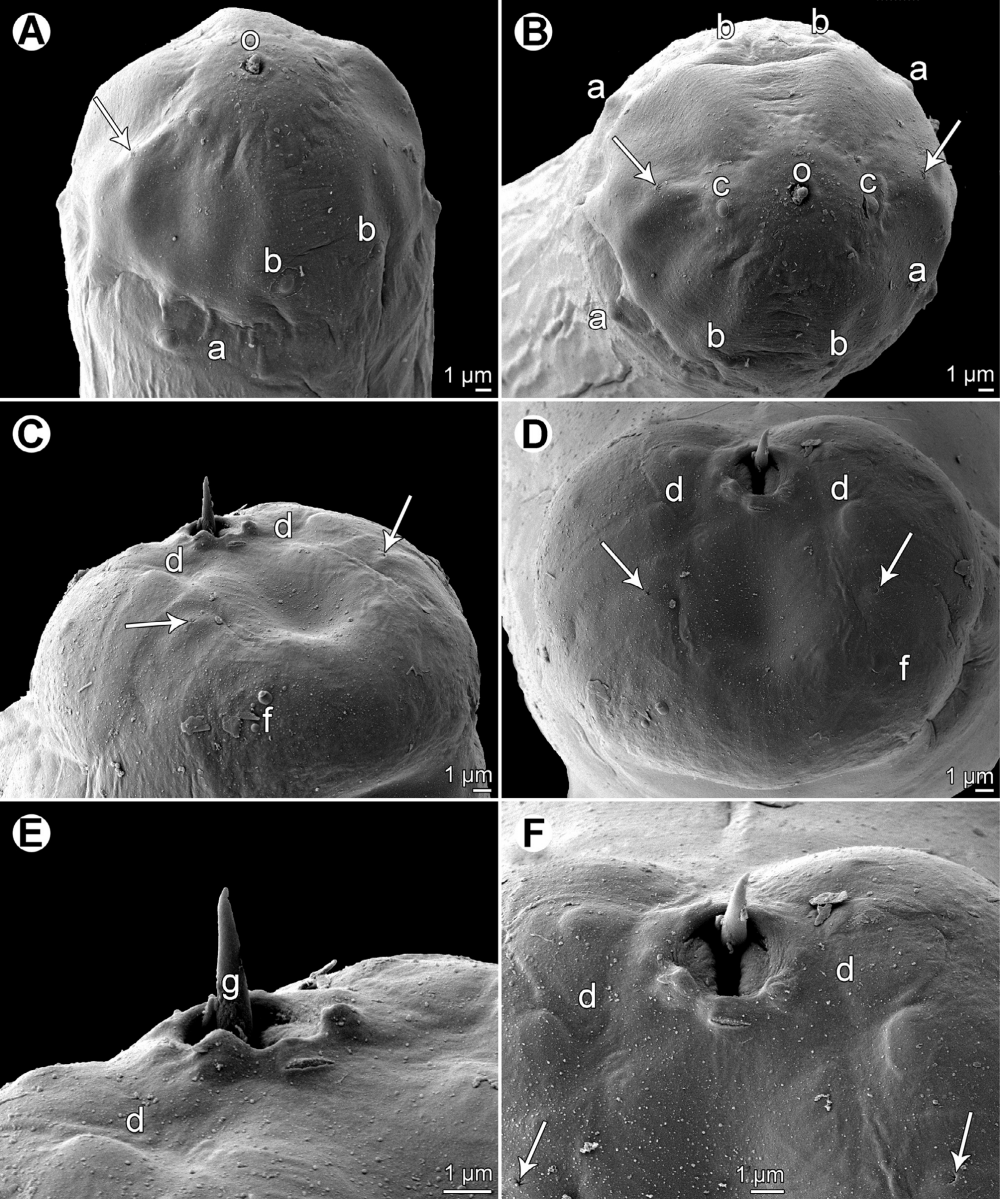
*Philometra selaris* n. sp. from *Selar crumenophthalmus*, scanning electron micrographs of male. A, B: Cephalic end, sublateral and apical views (arrows indicate amphids). C, D: Caudal end, subdorsal and apical views (arrows indicate phasmids). E, F: Region of cloacal aperture, lateral and apical views (arrows indicate phasmids). *Abbreviations*: a, submedian pair of cephalic papillae of external circle; b, submedian cephalic papilla of internal circle; c, lateral cephalic papilla of internal circle; d, pair of larger caudal papillae near cloacal aperture; f, caudal mound; g, gubernaculum; o, oral aperture.

Type host: Bigeye scad, *Selar crumenophthalmus* (Bloch) (Carangidae, Perciformes) (fork length 227–240 mm). Confirmation of identification by barcoding: one specimen (JNC313, GenBank KJ192346) had 100% identity; the other (JNC3126, GenBank KJ192345) had one base difference; this species is known to show molecular diversity [8].

Site of infection: Probably abdominal cavity (found in wash).

Type locality: Fish market, Nouméa City, New Caledonia. Fish were taken with mackerel nets within a few miles off Nouméa. Dates: 10 September 2009 and 25 November 2009.

Prevalence and intensity: two fish infected/seven fish examined using the “wash” method; one nematode specimen per fish.

Type specimen: Holotype (mounted on SEM stub) in the Helminthological Collection of the Institute of Parasitology, Biology Centre of the Academy of Sciences of the Czech Republic, České Budějovice (Cat. No. N–1043). The paratype specimen was destroyed during the SEM procedure.

Etymology: The specific name of this nematode relates to the genitive form of the generic name of the host.

### Description

*Male* (two specimens; measurements of paratype in parentheses): body filiform, whitish, 5.49 (5.26) mm long, maximum width at middle 48 (57). Cuticle smooth. Cephalic end rounded. Maximum width/body length 1:114 (1:92); width of cephalic end 30 (33), that of posterior end 30 (30). Oral aperture small, circular, surrounded by 14 cephalic papillae arranged in two circles: external circle formed by four submedian pairs of papillae; internal circle by four submedian and two lateral papillae (Figs. 8C, D, 9A, B). Small lateral amphids just posterior to lateral papillae of internal circle (Figs. 8C, D, 9A, B). Oesophagus 528 (390) long, maximum width 21 (21), forming 10 (7)% of body length, slightly inflated at anterior end; posterior part of muscular oesophagus overlapped by well-developed oesophageal gland with large cell nucleus situated somewhat posterior to its middle (Fig. 8A); anterior oesophageal inflation 36 (36) long, 21 (21) wide. Small ventriculus 3 (3) long, 6 (6) wide, present. Nerve ring, excretory pore and oesophageal nucleus 162 (159), 198 (210) and 423 (300), respectively, from anterior extremity. Testis not extending anteriorly to posterior end of oesophagus (Fig. 8A). Posterior end of body blunt, provided with broad U-shaped mound situated laterally and dorsally to cloacal aperture (Figs. 8E–G, 9C, D). Four pairs of genital papillae present, all near cloacal aperture: two pairs of larger, very flat and hardly visible papillae located on caudal mound (one adanal and one postanal pair) and one adanal pair of smaller papillae located between mound and cloacal aperture; additional pair of small, conspicuously elevated papillae present on posterior rim of cloacal aperture; phasmids situated somewhat posterior to papillae on caudal mound (Figs. 8E, 9C–F). Papillae indistinct under LM. Spicules short, slightly subequal, with somewhat expanded proximal and sharply pointed distal tips (Figs. 8F, G); length of larger left spicule 96 (87), representing 1.75 (1.65)% of body length, of smaller right spicule 93 (84). Length ratio of spicules 1:1.03 (1:1.04). Gubernaculum narrow, 75 (72) long, with anterior portion slightly bent dorsally; length of anterior bent part 39 (39), representing 52 (54)% of entire gubernaculum length; distal tip of gubernaculum pointed, smooth, with distinct dorsal reflexed barb (Figs. 8F, G, 9C–D). Length ratio of gubernaculum and larger spicule 1:1.28 (1:1.21). Spicules weakly sclerotised, whitish.

*Female*: Unknown.

### Discussion

The only philometrid species so far reported from *S*. *crumenophthalmus* is *Philometroides atropi* (Parukhin, 1966). It was originally described by Parukhin [29] based solely on females found in the abdominal cavity of the carangid fish *Atropus atropos* (Bloch & Schneider) off Vietnam (South China Sea, Gulf of Tonkin), and later the same author [30] reported it from the stomach wall of *S*. *crumenophthalmus* in the Red Sea. However, considering a relatively high degree of host specificity of philometrid nematodes, differences in the site of infection in the host and the fact that only nematode females were studied, it may well be that nematodes from *S*. *crumenophthalmus* were not conspecific with those from *P*. *atropi*. Since Parukhin’s material is probably lost (personal communication with Prof. A.V. Gaevskaya, Sevastopol), its reexamination is no longer possible.

It has been mentioned above that, besides gonad-infecting parasites, six nominal species belonging to four philometrid genera are known to parasitise carangid fishes: *Buckleyella buckleyi* Rasheed, 1963 from the mesentery of *Scomberoides* spp. off Pakistan and in the Red and South China Seas [29, 30, 34]; *Caranginema americanum* Moravec, Montoya-Mendoza & Salgado-Maldonado, 2008 from subcutaneous tissues of *Caranx hippos* (Linnaeus) in the Gulf of Mexico [11, 12, 24]; *Philometra grandipapillata* Moravec & Bakenhaster, 2010 from the subcutaneous tissues of *C*. *hippos* in the Gulf of Mexico [11]; *P*. *tauridica* Ivashkin, Kovaleva & Khromova, 1971 from the abdominal cavity of *Trachurus mediterraneus* (Steindachner) in the Black Sea [5]; *Philometroides atropi* (see above); and *P*. *seriolae* (Ishii, 1931) from the musculature of *Seriola quinqueradiata* Temminck & Schlegel off Japan [4, 25, 28, 33].

Most of these six species are known solely from their females, whereas conspecific males have been described only for *C*. *americanum* and *P*. *tauridica* [5, 12]. In contrast to both of these species, philometrid males in the present material from *S*. *crumenophthalmus* are markedly larger (body length 5.3–5.5 vs. 3.1–3.3 or 1.5–3.0 mm) and their gubernaculum is also longer (72–75 vs. 48–51 or 30–58 μm); moreover, they were collected from fishes belonging to a different genus (*Selar* vs. *Caranx* or *Trachurus*) in a distant geographical region (South Pacific vs. North Atlantic or Mediterranean region). Males of the remaining four philometrid species from carangids are not known and, consequently, cannot be compared with those of the present material; however, these species can be separated based on the different genus of their type host and the geographical distribution (see above). Therefore, we consider the two philometrid males from *S*. *crumenophthalmus* to represent a new species, *P*. *selaris* n. sp. The allocation of this species to *Philometra* is provisional; present philometrid genera are mostly based on the morphology of gravid and subgravid females [1, 5, 9, 15, 34], whereas males of some genera (e.g. *Caranginema*, *Philometra* and *Philometroides*) are unidentifiable to the generic level.
